# Long acting injectable buprenorphine: Perspectives from service-users, staff and stakeholders^☆^

**DOI:** 10.1016/j.dadr.2025.100328

**Published:** 2025-03-29

**Authors:** Rebecca Fish, Céu Mateus, Hannah Maiden, Euan Lawson, Mark Limmer

**Affiliations:** a Division of Health Research, Lancaster University, UK; b Lancaster Medical School, Lancaster University, UK

**Keywords:** Opioid agonist therapy, Long acting injectable buprenorphine, Recovery, Harm reduction, Qualitative

## Abstract

**Introduction:**

Long-acting injectable buprenorphine (LAIB) is a relatively novel pharmacological treatment for people with opioid dependence. Despite growing qualitative evidence, there is limited research on practitioner insights, and effectiveness of LAIB in a community setting.

**Methods:**

Thirteen service-users (11 currently prescribed LAIB), 6 practitioners, and 4 stakeholders (public health workers) took part in semi-structured interviews (n = 23) to glean their perspectives on LAIB. They were recruited through a community drug treatment service in the NW of England. The interview schedule was informed by previous literature and co-produced with a peer worker with lived experience of drug recovery treatment. Transcripts were analysed thematically by the research team.

**Results:**

Four major themes were identified from the interviews: A change of focus; challenges; wrap-around support; and target groups.

**Discussion:**

Our findings support existing evidence around the individual benefits to service-users such as changes to lifestyle and reduction of stigma, as well as challenges such as the need for wrap-around support and accessible information. We found that commissioning considerations such as geographical inequalities and the need for multi-service collaboration are important in this setting.

**Conclusions:**

LAIB treatment works well for many people in a community context that offers significant wrap-around support to service-users. The novelty of this research lies in bringing together the views of practitioners and stakeholders as well as treatment/service beneficiaries in evaluating the introduction of LAIB in a community service.

## Highlights

• Long-acting injectable buprenorphine (LAIB) is a new opioid replacement treatment.
• LAIB can have a positive effect on lifestyle, clarity of mind, and reduced stigma.
• There is a need for more information, and well-funded wrap-around services.
• There are differences in opinion about offering LAIB in a harm reduction context.

## 1 Introduction

Methadone and buprenorphine are effective pharmacological treatments for people with opioid dependence, retaining people in treatment and suppressing illicit opioid use (Mattick et al., 2003), and are on the World Health Organization Model List of Essential Medicines (World Health Organization, 2023). In the UK, they are deemed cost-effective and are recommended by the National Institute for Health and Care Excellence (NICE) in the treatment of heroin dependence (National Institute for Health and Care Excellence, 2007).

There are many factors that clinicians and patients will consider when deciding on the best medicine. There is evidence that patients on methadone are more likely to be retained in treatment compared with buprenorphine (Degenhardt et al., 2023). However, evidence is accumulating that buprenorphine is less likely to be associated with the risks of fatal overdose (Tanz et al., 2023), particularly when initiating treatment in the induction phase. However, UK guidance states that there remains insufficient evidence to justify recommending one drug over the other (Department of Health, 2017) and promises updates to include further guidance in 2025 (Department of Health, 2024).

Supervision of buprenorphine remains challenging when compared to liquid methadone, as the tablet takes time to dissolve and there is a risk of diversion (Lofwall et al., 2014). Therefore, new forms of buprenorphine were introduced to the market – either as films or tablets which rapidly dissolve, or in long-acting injectable form administered as a monthly or weekly injection.

The National Institute for Health and Care Excellence (2019) conducted an evidence review in 2019 on long-acting injectable buprenorphine (LAIB). At that time, the preparation available was Buvidal©, given weekly (8 mg, 16 mg, 24 mg) and monthly (32 mg, and 64 mg, 96 mg, 128 mg and 160 mg). This guidance states that this treatment may be an option for people where there may be a risk of storing medicines at home and those who experience barriers in accessing daily supervised medication, including those in custodial settings. This treatment was introduced in 2019 in the UK, and prescribing levels are growing but remain low (Rolland et al., 2024) mainly due to high relative cost. Current Department of Health recommendations (Department of Health, 2024) state that the patient could be switched to LAIB if they are observed to be not benefitting from unsupervised buprenorphine or methadone dosing (with more guidance to follow in 2025).

At the time of the 2019 NICE review there was just a single study (Lofwall et al., 2018). This was a double-blind double-dummy study and demonstrated that LAIB was non-inferior to sublingual buprenorphine. The initial phase 3 LAIB trial was a randomised double-blind controlled trial and it was conducted across 36 treatment centers in the USA (Haight et al., 2019). It showed that the monthly dosing regime was effective in retaining people in treatment (See also Williams and Saima, 2023). A further report on this phase 3 trial found improvements in patient-centered outcomes (Ling et al., 2019). It must be noted that the control group was placebo in these studies, which has various practical and ethical implications (see Nunes et al., 2016; Strickland and Stoops, 2018). Later studies in Australia have demonstrated positive treatment retention as well as reductions in non-prescribed opioid use, and improvements in employment and quality of life (Larance et al., 2020, Farrell et al., 2022). In these studies, the retention rates were high – but these were individuals who were already established in treatment and stable on 8–32 mg sublingual buprenorphine, and who had expressed an interest in receiving LAIB.

Existing quantitative literature demonstrates increased quality of life and satisfaction with treatment (Lintzeris et al., 2022, Melichar et al., 2022, Farrell et al., 2022). Ling et al. ’s (2019) US based study of 389 participants receiving depot buprenorphine (n = 389) versus placebo (n = 98) demonstrated significantly greater health-related quality of life scores (mental and physical component scores at P = 0.002). They found that satisfaction was significantly higher than placebo, employment increased by over 10 %, and participants had significantly fewer hospital days per person-year observed.

When reviewing recent international qualitative studies into service-user experiences of LAIB using various literature databases such as PubMed and BioMed Central, our review found perceived benefits to be increased convenience to travel and work, improved relationships, and reduced stigma due to there being no need for supervised daily dosing (Jaffe et al., 2024, Martin, 2021, Neale and Strang, 2024, Allen et al., 2023, Barnett et al., 2021). Common concerns expressed were around side effects, the return of emotions due to clarity of mind, lack of daily routine, and reduced contact and disconnection from healthcare services (Martin, 2021, Neale et al., 2023, Nordgren et al., 2024). Barnett et al. (2021) recruited 30 participants from various sites in Australia, finding that the positive benefits included avoiding stigma experienced at pharmacies/clinics and more time to engage in activities such as travel or work. However, some people felt constrained in their control and flexibility of dosing.

A 2021 peer-led evaluation of LAIB in Wales elicited the views of 94 participants (75 % male) (Gwent Drug and Alcohol Service, 2021). They reported rebuilding their lives, getting jobs, reconnecting with family members, and fewer cravings, as well as lower anxiety levels and reductions in offending. Negatives were reported by those who felt unprepared, receiving insufficient support or information. Some participants described experiencing overwhelming emotions, multiple side effects, and difficulties with finding new ways of living.

Emotional responses to LAIB treatment are common in the existing literature, for example Matheson et al. (2022) investigated the views and experiences of people in Scotland who were homeless. Participants described the experience of opiate agonist therapy (OAT), comparing the “numbing/comforting” effect of methadone versus the clear-headedness and “awaking of emotions” with LAIB (see also Parsons et al., 2020). Further, Martin (2021) surveyed 53 participants in Scotland, finding that the LAIB retention rate in this sample was higher than the median 6-month retention for either methadone or sublingual buprenorphine. The conclusion from this study was that the clarity of mind associated with buprenorphine had clearly not been a barrier to treatment retention. Martin observes that their participants maintained a level of stability that they may not have previously achieved on either methadone or sublingual buprenorphine.

Qualitative studies with stakeholders, staff and service-providers are less common. Nordgren et al. (2024) interviewed stakeholders, staff and physicians in Sweden. Their participants considered LAIB as a valuable treatment option particularly for patients with low treatment adherence, but had concerns about power dynamics and loss of treatment alliance. Magel et al. (2024) study with stakeholders and service-providers in Canada demonstrates the potential for LAIB to provide greater choice and flexibility, but service providers and staff felt held back by system-level regulations that negatively influenced power dynamics, for instance during period of non-adherence. Finally, Reddy et al. (2024) focus group study in the USA with outpatient providers found uncertainty about patient candidacy and concerns about high cost. The current study is the first to feature the perspectives of both service-users and stakeholders/staff.

## 2 Context

Blackpool is a coastal region in North West England with a population of around 140,000 people. It is the most deprived local authority in England with more than a quarter of children living in low-income households. People in Blackpool experience the lowest life expectancy in England (Office for Health Improvement and Disparities, 2024), and there is persistently high drug related harm, with estimated prevalence of drug use over three times the national average (JSNA Blackpool, 2024), and drug related death rates being almost four times the national average (Office for National Statistics, 2023). LAIB was introduced in community treatment services in Blackpool as part of the Drug Harm Reduction Strategy 2020–2022 delivery plan, with the aim that people who use drugs are ‘offered evidence-based treatment and holistic support to give them the best chance of achieving recovery’ (Blackpool Council, 2019:18).

This qualitative study formed part of an NIHR funded mixed methods evaluation of the use of LAIB in the context of Blackpool with beneficiaries of a community-based third sector council funded drug treatment service for people who are opioid dependant. The study also includes a quantitative element using longitudinal self-reported data (forthcoming).

The research question was: What are the experiences, (including drug treatment, health and wellbeing, and social), of people started on LAIB in Blackpool?

## 3 Method

Recruitment and ethics: Ethical approval was granted by Lancaster University Faculty of Health and Medicine Research Ethics Committee. Recruitment of service-users took place through drug treatment and recovery services and a third sector lived experience organisation, and was purposive, requiring participants with present or past experience of LAIB. Service-users were informed about the study by their keyworker and given the link to the information sheet and consent form for them to consider signing. The sheet informed potential participants that participation was voluntary and anonymous and that the service they received would not be influenced by their decision about taking part. When the online consent form was signed and contact information provided, this flagged the researcher to contact the potential participant. Staff and wider stakeholders were purposively recruited using targeted email requests using snowball sampling, that contained the link to the information sheet and consent form for them to sign. At the beginning of the interviews, each participant was reminded of their right to withdraw their interview data over the subsequent two weeks, and that their data would be confidential and anonymised, unless a risk of harm was communicated during interview. Personal data were kept separate from interview data and deleted after interview with the exception of consent forms which were kept on encrypted survey software.

Thirteen service-users, 6 staff (including clinical, treatment and nursing/prescribing staff) and 4 stakeholders (public health workers, including regional and national commissioners and policymakers, and public health council workers) agreed to be interviewed (see Table 1). Service-users consisted of 9 women and 4 men, with the majority being in the 40–49 age bracket. Most service-users had been prescribed LAIB for 3–12 months, and 2 had successfully completed treatment. Interviews lasted between 25 and 75 minutes. Most interviews were held online using encrypted video calls, and 4 service-user interviews were held in person at a private room in the community service.

**Table 1:** Demographic details of interviewees.

**Category**	**Details**	**Number**
**Service-users**	Interview participants 1–13	
**Gender**	Women	9
	Men	4
**Age**	30–39	1
	40–49	8
	50–59	4
**Duration LAIB**	0–3 months	3
	3–6 months	4
	6–12 months	5
	12–24 months	1
	No longer prescribed LAIB	2
**Staff/Stakeholder**	Interview numbers 1-10	
**Gender**	Women	6
	Men	2

The interviews were in a semi-structured format, with guiding questions and prompts decided beforehand informed by the literature review, and in consultation with a researcher with lived experience of OAT. Service-user participants received a £ 15 shopping voucher for their time. Interviews were recorded and transcribed using encrypted software, with the researcher performing anonymisation and transcription checks.

Analysis: The data was analysed using a critical realist approach, which seeks to take into account the exploratory (experiences) and the explanatory (causal relationships) (see Fryer, 2022; Jaffe et al., 2024). The researcher began the coding themes using the analytical thematic analysis steps described by Braun and Clarke (2006). This involved generating initial codes using the qualitative analysis software NVIVO, then defining themes by taking notice of the connection and patterns between the codes. The research team consisting of all 5 authors met a number of times to discuss the analysis, and revisited the broad themes in an iterative process to develop subthemes with each reading of the transcripts. This resulted in subsequent revision of the generated themes until the data had been rigorously explored and consensus was met, with members performing a coding check on the final complete matrix.

The following section will explore each analytical theme, providing representative quotes for illustration that capture the nuanced perspectives of each group. It is highlighted where there are conflicting quotes within that theme. The results are presented below, organised into major and minor themes, and all names are pseudonyms.

## 4 Results

The thematic map demonstrated much agreement between service-users and staff/stakeholders, particularly when discussing service-user experiences and concerns. Staff/stakeholders additionally considered operational, overarching issues. There were some diverging opinions which were revealed during the thematic revisions, in particular around which service-users should be offered LAIB. Please refer to Tables 2–5 for service-user and staff/stakeholder illustrative quotations for each theme.

### 4.1 Major theme: a change in focus

Within this theme were descriptions about how LAIB has enabled people to focus on other aspects of daily life beyond accessing illicit drugs, as detailed in Table 2.

**Table 2:** A change in focus.

**Group name**	**Quote**
Service-users	1. David: It stops the craving, it stops the initial wanting it. It just stopped everything about heroin for me. 2. Sarah: I can go during the week while the kids are at school and whatnot. It has made life much easier in that respect. It's done and it's forgotten about. I've got a decent job. I've just been promoted as well. So everything is going really well. 3. John: I was the worst of the worst, I'll be honest. And it made me want to become a better person, control my temper. Think how I treat people now. 4. Frank: I've applied to go to college in September. So higher education, a bit of volunteering and employment hopefully, holidays. And my family's coming back in my life. I just want a normal, happy life. 5. Sue: It just gives me a bit of a spring in my step. You just feel completely different, more aware of everything. I feel healthier. 6. David: At the moment, I've got a lot of problems at home. But I know for a fact if I weren't on [LAIB] I'd be bang at it, taking heroin and crack cocaine. 7. Ian: There was no rattle, so I felt quite comfortable just to take it a day at a time, and just connect to people and go to meetings. I was really happy to have the opportunity to be prescribed it. 8. Alison: I had to sink [methadone] in the chemists. Yeah, it was horrible. And, everyone looked down on you because there's a special window where all the addicts go. Some of them are absolutely wasted when they're there. And when you're in the queue for your meds they shout your name, and I don't want them to know my name. 9. Eve: When I was on the methadone, as much as it kept me from being poorly, I was sick of that life but I had no power to do it by myself.
Staff/stakeholders	1. Carrie: I like the idea that it's got the blocker in it, it dissuades people from using illicit substances. And I think that that's a good thing. As long as there's a plan in place to manage the associated risks. 2. Maya: We don't realise as service providers, how much power we have. The role already has such a big power play. [LAIB] empowers people to carry on with their lives, without having that fear of negotiation for a script or to attend a pharmacy.

Some participants mentioned that LAIB stops the cravings of heroin, and indeed partially blocks the potential effects, as mentioned by service-user David, *‘It stops the craving, it stops the initial wanting it. It just stopped everything about heroin for me’* (see also Carrie in staff quote 1). Lifestyle impacts included being able to fit treatment around employment and childcare, as mentioned by Sarah (service-user quote 2). Many service-users remarked on the positive change in their family relationships, future plans, and sense of self (see service-user quotes 3 and 4). Alongside these positive changes, improvements in health, resilience, and service engagement were described (see service-user quotes 5–7).

Avoiding the stigma of supervision was mentioned as a significant benefit by all groups, including the empowerment and agency that this can bring (see Alison, service-user quote 8, and Maya in staff quote 2). Eve (service-user quote 9) was encouraged to try LAIB so that she could access a recovery home placement. She felt empowered towards abstinence due to this.

This data demonstrates the experience of a breadth of positive life changes for people who use LAIB. Importantly, most participants mentioned that the benefits were realized alongside the wrap-around service input they had received.

### 4.2 Major theme 2: challenges – not the ‘miracle’

Every participant mentioned challenges of LAIB treatment as outlined in Table 3. Discussion about challenges treatment fell into 2 subthemes – the challenges experienced by individual service-users; and commissioning and service provision measures that are needed to allow the real benefits of this treatment.

**Table 3:** Challenges: ‘not the miracle’.

**Group Name**	**Quote**
Service-users	1. John: At first, it was hard getting to grips with them [emotions] coming back. And guilt, that was a big one, that coming back. 2. Ian: I did use other drugs. Predominantly weed, benzos and the odd crack pipe. The first month was hard because I was all over the place. There was people where I live, everyone uses, it’s the lifestyle. 3. Emma: At the minute because I'm so stable I don't want to take that away. The last thing I want to do is take that away, and then end up back where I was. 4. Sarah: My biggest fear at the minute, is coming off everything because of how it makes me feel to withdraw. I've gone through it three times [with other treatments], and been really poorly the last time. 5. Sarah: They gave me the booklet at the time told me to have a read through that at my leisure. I was warned about getting lumps from the oil gathering. And that was it really. I didn't get much to start with to be honest, there wasn't much more information they could give me with it all still being new. 6. Frank: It were a friend who told me about it. So I thought I would go into services. I saw a nurse and she asked how much I were using and how often and that. 7. Julie: My partner did keep saying, ‘Why don't you go on the injection?’ But I had it in my head that if I only have an injection once a week I'm gonna be rough because I've not had a daily injection. That's how l thought at the time. 8. Emma: I've got a bad legs, I've got a bad back, and you're very limited to, as to what painkillers you can use.
Staff and stakeholders	1. Carrie: It does just seem to leave a lot of lumps on, especially people that are built slightly. That worries me a bit. The people we have, they tend to be quite slight build, especially the ladies. We try to go for the tummy area when that happens. 2. Kelly: Some people I don't think are ready for it because they do work out pretty quick that the opiate receptors are blocked. And their choice is taken away, isn't it? I think some like to keep that choice. 3. Maya (Stakeholder): A lot of people will also get information from their peers as to how they feel on the medication. I think that's a very strong form of information for people wanting Buvidal. 4. Carrie: I go through the booklet with them. I go through the [other medications] that don’t work. It's an opiate blocker, you see. 5. Joanne: What makes everybody a little bit anxious is the push from the prisons, putting people on Buvidal, and then that anxiety of - will they be able to get it when they're released from prison in the local authority. 6. Zara (Stakeholder): It's taking out the nursing time when we probably need our nurses to think about the physical health of the people. So there's an opportunity cost as well as the drug cost. 7. Kelly: If they're on the methadone script, at least they're seen, either daily or every other day. Once they've come in for the Buvidal they may just think they’re alright, and then no one sees them for a week or a month. 8. Jane: For some people, residential rehab will be the best option for them. But they can’t, they're not clean. They're not off drugs, because they're taking Buvidal. 9. Maya: We have to know what's happening in their lives, because a lot of their socialising, a lot of the structure to their day used to be around collecting their medication, seeing their key worker, going to a club, meeting people. So once you give somebody Buvidal, you're actually cutting off that link from the pharmacist, and they might not see their key worker that often. 10. Maya: You can't expect someone to engage in therapy when they're not even housed, or they're living in squalor. Also, there should be access to health care that suits the needs of people who use substances because they have difficulty getting pain management or accessing services. It's all interlinked. 11. Carrie: I've had consultants, doctors, nurses phoning me and saying, 'What is this drug? Is this a pain relief?' They don't understand it. And that worries me. You know, I think they won't give them pain relief.

### 4.3 Subtheme: Individual challenges

Commonly raised challenges for individuals included the return of emotions, recognising past behaviour and trauma, as explained by John (service-user quote 1). Many people tried other substances when they first started on the LAIB treatment. This was often related to the void in their lives left by no longer using opioids (see Ian, service-user quote 2).

Table 3 contains quotes related to this theme. Side effects, including issues with mental health, digestion, sleep, and lumps in the skin were commonly mentioned by staff such as Carrie (staff quote 1), and Ian (service-user quote 2). Further, the lack of choice/control due to the partial blocking action of LAIB featured in observations by nursing staff such as Kelly (staff quote 2). Although there was mention of challenges with side effects and emotional issues, these were often short-term, and participants mentioned that services generally had systems in place to mitigate them.

In terms of progressing to the point where they no longer took LAIB, most people who were still taking it were concerned about disturbing their stability, and many stated that they were unsure what to expect (see service-user quotes 3–4).

Related to this, many participants felt that there was not enough accessible information available. Service-users often told us they had been given a booklet that was formal and difficult to read (see Sarah, quote 5). Many service-users (e.g. Frank, quote 6) had accessed informal information via peers and some staff expressed concern about this (e.g. Maya, staff quote 3). Further, Julie (service-user quote 7) eventually decided to try LAIB after initially being worried about withdrawal, highlighting the need for more general information and awareness. Nursing staff also commented on the lack of accessible information, which meant they were required to spend time providing information and reassurance, with implications on their workload (e.g. Carrie, staff quote 4).

### 4.4 Subtheme: service and commissioning issues

Staff and stakeholders mentioned challenges including variations in cost and availability in different areas, and high use of resources in terms of releasing specialist staff for administering treatment (see Joanne and Zara, staff quotes 5 and 6). Some staff had concerns that LAIB may cause people to be less engaged with services, with implications on their recovery or progression through services. The need for housing and health services were concerns for some people and discussion tended to be around a general lack of knowledge of LAIB (see staff quotes 7–10).

Some participants (e.g. service-user quote 8 and staff quote 11) mentioned a lack of knowledge about LAIB and pain relief in healthcare services. Participants therefore articulated a variety of commissioning challenges that went beyond drug treatment services.

### 4.5 Major theme: wrap-around support

For the benefits of LAIB to be realised it had to be considered in the context of wider wrap-around support, as demonstrated by quotes in Table 4. Some people mentioned the possibility of a dip in mental health during early days of taking LAIB, (see Carrie, staff quote 1). Additional support was extremely important to all groups of participants, and people often mentioned having the clarity and space to engage more meaningfully with support. Service-users had access to various psychosocial activities including support groups, lived experience groups, psychological therapies, and daily activities - and they frequently mentioned the significance of these (see Eve and Rachel, service-user quote 2–3 and Mick, staff quote 2).

**Table 4:** Wrap-around support.

**Group name**	**Quote**
Service-users	1. John: [The groups] help you get to the root cause of why, and how you should feel appropriately about stuff. It reinforces that you're not a bad person, you just made bad decisions. 2. Eve: When other people are talking about their life, we can we all relate to one another, it's very positive. The feedback and help you get, if you don't understand something the guy who runs it explains it in easier ways. 3. Rachel: People who have been on the same drugs as you and you're looking at them, they're telling you that they're five years clean and the way they got clean, doing meetings, staying connected, keeping yourself busy. Being around the right type of people, that has been a game changer for me.
Staff/stakeholders	1. Carrie: I've noticed with men, there seems to be a decline with some in their mental health. That does seem to be a pattern. 2. Mick: [We need to take into account] adverse childhood experiences, and current experiences, helping people to develop that better sense of self. I think it's all about developing supportive relationships, in which people can feel they can cope and be supported in dealing with life’s difficulties. 3. Larry (Stakeholder): The stigma in treatment is as corrosive as the stigma people experience outside of treatment. We do quite a lot of stigmatising things, and we think it's us trying to do the right thing for them. When actually, you know, it's the right thing for us. 4. Maya: I think [LAIB] has a role but it might not be suitable for everyone. And we need to acknowledge that. It's not the miracle, it's still the same chemical. What’s important is accessibility for psychosocial interventions and reducing the stigma of getting support. 5. Larry (Stakeholder): By engaging people we can work on motivation, we can get people to reflect on their current situation, without telling them that they should be considering recovery. The medication can be used to support their own treatment goals. And by having them engaged, we can consider what next?

Having contact with people with lived experience in support groups and activities was also a key theme, with all service-users highlighting the importance of this. Staff and stakeholders discussed future priorities for service provision in this respect, such as the need for reducing stigma and using trauma informed approaches. Staff and stakeholders were keen to give a nuanced view of the treatment, making sure that services do not use LAIB as a way to provide light-touch support, and reiterating the need for psychosocial support (staff quotes 3–5). This theme therefore demonstrates the importance of person-centered wrap-around support for people being prescribed LAIB.

### 4.6 Major theme: target group

There was divergence of opinion within this theme as shown in Table 5. The majority of service-users argued that LAIB is best for someone who is accessing psychosocial support and is ready to stop using drugs, as LAIB is a partial agonist and takes away the choice to continue using. Power and agency were often mentioned as part of this discussion (see Sue and Ian, service-user quotes 1 and 2).

**Table 5:** Target group.

**Group name**	**Quote**
Service-users	1. Sue: I think the person’s got to be ready, if that person isn't ready then it's pointless hoping to go on [LAIB]. If they want to still use, it's pointless giving them something that is going to last a week or a month. Nobody should be forced onto it because previously I was being forced to go on it, that's why I kept sabotaging and using. So the person most definitely has to be ready. 2. Ian: Someone who wants it. Someone who's asking questions, someone who's saying, ‘Look, I need help, I can't do this on my own’ - then you can start suggesting alternate avenues. 3. Sue: I’d been on methadone for years. You feel sluggish on methadone and this gives me energy and that's quite an achievement. With methadone, I'd wake up and I knew by nine o′clock I had to go and get it because I was feeling unwell. This [LAIB], you're more awake, aware of your feelings, you're not getting sedated anymore. 4. Ian: I started being more consistent with my appointments. I was able to go to the gym, I was feeling well. I cried watching telly! But if I was on methadone I wouldn't even feel anything. With crack, it's heightened senses, heroin dulls it. With Buvidal it just makes you feel clean, like you're normal.
Staff/stakeholders	1. Kelly: People that are street homeless, the ones who would find it difficult going to a chemist every day, or people who are at risk of say being cuckooed or manipulated, at risk of financial abuse. 2. Larry (Stakeholder): I think people could just take a break from using rather than LAIB being seen necessarily as a long-term intervention: ‘Why don't you have a few weeks or a few months off, give yourself a bit of a break, see what that's like?’ I'm not sure that it's been sold in that way. I think we could refine the messaging far better, to attract people in even the short-term, and get them into better health interventions during that period. 3. Larry (Stakeholder): You bring in people that either don't come in or don't stick in treatment, meeting this greater unmet need. And ultimately, that means drug related deaths are likely to be affected positively.

Some practitioners and stakeholders argued that LAIB should be made available to people who otherwise would not engage with services (e.g. Kelly, staff quote 1), however, two stakeholders discussed the opportunity for using LAIB in a harm reduction sense, as a way to reduce drug use or give the person a break, during which they might engage further with services (see for example Larry in stakeholder quote 2).

Comparisons with other OATs were generally favourable to LAIB. People highlighted the energy and clarity they experienced (Sue and Ian, service-user quotes 3–4), whereas staff and stakeholders appreciated the ability to offer choices in terms of treatment, and the potential for LAIB to reach people who might not otherwise engage with services, therefore addressing health inequalities. This relates to the notion of the target group, and may have implications on treatment outcomes (see Larry in stakeholder quote 3).

This theme demonstrates a lack of clarity regarding who should be offered or prescribed LAIB, and at which part of their recovery process. Certainly, having a choice of medication available was appreciated by many respondents.

## 5 Discussion

Our study provides an insightful and novel contribution to the literature by examining both service-user and health worker perspectives in a community drug service, and found that LAIB enables people to focus on positive change instead of daily drug use, and can support improved resilience and reductions in stigma. However, a range of individual and commissioning challenges with the treatment are apparent, as well as the need for a holistic package of care alongside the medication. Our data demonstrates uncertainty about the cohort(s) for whom LAIB benefits most and therefore the need for more in-depth research in this area. Despite the increasing number of recent articles exploring the views of people prescribed LAIB, this is the first to feature the perspectives of beneficiaries alongside practitioners and stakeholders in a community setting.

In agreement with existing literature, our study has established that people prescribed LAIB find it convenient in terms of childcare and employment, feel clearer of mind, and experience few withdrawal effects (Martin, 2021, Allen et al., 2023). Participants from all categories in our study pointed out the importance of wrap-around support in the form of psychosocial interventions, therapeutic relationships, daily planned activities, and peer contact to ensure the success of LAIB treatment (see also Blawatt et al., 2023; Friedmann et al., 2023; Melichar et al., 2024; Neale et al., 2023; Neale and Strang, 2024). The service-users in this study had access to various psychosocial activities including support groups, lived experience groups, psychological therapies, and daily activities. The importance of wrap-around support was key to the perceived success of LAIB treatment.

Due to this treatment pathway being relatively new, the need for information was a key concern for service-users (Ward, 2023) who were often accessing information through peers. They stressed the importance of accessible information from the perspectives of those who have experience of the treatment, including what to expect when starting on LAIB, side effects, and challenges of reducing dosage. This aligns with findings from other studies that emphasise how information provided by services may be perceived as biased or coercive (Neale et al., 2019a, Neale et al., 2019b). Neale et al. (2019a) found that participants decided to try LAIB after receiving information about LAIB’s impact on illicit drug use and recovery, and its perceived effectiveness (see also Tompkins et al., 2019). Our findings add to this by elucidating on the need for more co-produced and experiential evidence and information.

To facilitate information exchange, Holloway et al. (2022) recommends face-to-face consultations, as well as access to experiential information via peers. The participants in this study valued verbal information from staff as it was tailored and accessible, however they also required information about what to expect, that could be taken away and referred to at different stages of LAIB treatment.

The LAIB commissioning considerations that emerged in our study include: geographical inequalities in availability, pathways to rehabilitation, how to facilitate engagement with other services, and a lack of clarity on concurrent pain management. The need for specialist staff to administer treatment also has commissioning implications. These findings are novel and arise from this study using a combined sample to elicit multiple perspectives. We found that during consultations, practitioners introducing a new treatment such as LAIB were providing information and support. This aligns with a study by Nordgren et al. (2024), who noted that staff need to spend extra time giving information to patients so they know what to expect. Their participants also experienced increased concern about power dynamics and loss of therapuetic alliance due to the potential for reduced contact. Concerns about power and agency were prevalent among staff and stakeholders in the current study, with some staff considering LAIB to allow the service-user more agency, and some service-users reporting a lack of agency due to coercion (see also Johnson et al., 2022). Taken together, these findings emphasise that LAIB is not just an alternative treatment choice in the formulary, but that its delivery requires consideration in terms of wider service and system issues.

An important and novel finding was the discrepancy in opinion on who LAIB should be offered to. Service-users and some practitioners commented that it is important that a person started on LAIB has made a decision that they want to stop using drugs, whilst some stakeholders suggested offering LAIB to people as a primary, harm reduction step. McKeganey et al. (2004) discuss the requirements of people who use drugs when they contact services, observing that almost half of all people do not have the goal of abstinence at this point. This difference in perspective may stem from the complexities of concepts of ‘recovery’ and the lens through which this is seen (Neale et al., 2023, Westover and Mendonca, 2025). The implications of opinions about who is the ‘ideal LAIB patient’ may skew any outcome measures as well as service engagement if only people who require abstinence are offered treatment. Understanding which cohort(s) of people who use drugs may benefit from LAIB as opposed to other forms of OAT at different points in treatment requires further, outcome-focused research.

### 5.1 Limitations

The participants from this study were a small sample taken from a single community in Blackpool and differing perspectives may have emerged elsewhere. Whilst recruitment was purposive, we found it difficult to access people who had discontinued LAIB (Parkin et al., 2024), so our findings may be weighted towards service-users with more positive experiences. We therefore recommend further in-depth study of the use of LAIB in various settings including custodial, outreach, and residential services. In addition, further study of people who have declined or discontinued LAIB would be valuable.

## 6 Conclusion

Our study shows that LAIB treatment works well for many people in a community context that offers significant wrap-around support to service-users. It demonstrates the importance of eliciting the views of practitioners and stakeholders as well as treatment/service beneficiaries when evaluating the introduction of new medication regimes.

## CRediT authorship contribution statement

**Fish Rebecca:** Writing – original draft, Writing – review & editing, Methodology, Investigation, Formal analysis, Data curation. **Maiden Hannah:** Supervision, Methodology, Formal analysis, Conceptualization, Writing – review & editing, Funding acquisition. **Mateus** **Céu:** Writing – review & editing, Supervision, Methodology, Formal analysis, Funding acquisition, Conceptualization. **Limmer Mark:** Writing – review & editing, Project administration, Methodology, Funding acquisition, Conceptualization. **Lawson Euan:** Writing – review & editing, Methodology, Formal analysis, Conceptualization, Funding acquisition.

## Declaration of Competing Interest

The authors declare the following financial interests/personal relationships which may be considered as potential competing interests: Céu Mateus reports financial support was provided by National Institute for Health and Care Research. The other authors declare that they have no known competing financial interests or personal relationships that could have appeared to influence the work reported in this paper.
